# Meditation and Its Mental and Physical Health Benefits in 2023

**DOI:** 10.7759/cureus.40650

**Published:** 2023-06-19

**Authors:** Aneeque Jamil, Sai Dheeraj Gutlapalli, Marya Ali, Mrinal J. P. Oble, Shamsun Nahar Sonia, Sherie George, Srushti R Shahi, Zahra Ali, Abdelrahman Abaza, Lubna Mohammed

**Affiliations:** 1 Internal Medicine, California Institute of Behavioral Neurosciences & Psychology, Fairfield, USA; 2 Internal Medicine Clinical Research, California Institute of Behavioral Neurosciences & Psychology, Fairfield, USA; 3 Psychiatry, California Institute of Behavioral Neurosciences & Psychology, Fairfield, USA; 4 Internal medicine, California Institute of Behavioral Neurosciences & Psychology, Fairfield, USA; 5 General Medicine, California Institute of Behavioral Neurosciences & Psychology, Fairfield, USA; 6 Medicine, California Institute of Behavioral Neurosciences & Psychology, Fairfield, USA; 7 Medicine, California Institute of Behavioral Neurosciences & Psychology, California, USA; 8 Pathology, California Institute of Behavioral Neurosciences & Psychology, Fairfield, USA

**Keywords:** meditation and physical health, meditation and immunology, meditation and genetics, meditation and mental health, meditation

## Abstract

This article discusses the power of meditation and how beneficial it is for the body. Magnetic resonance imaging (MRI) has shown many positive brain changes and improved several brain functions. Meditation has several benefits improving the immune system and inflammatory processes by decreasing cytokine; appropriate telomere shortening also has helped healthy aging. Regarding physical health, meditation has been beneficial in various multi-factorial diseases like diabetes, hypertension, and fibromyalgia. It has also helped bring down blood cholesterol levels and increase high-density lipoproteins (HDL) levels. Improvement was also seen in systolic and diastolic blood pressure. Mental health is another aspect influenced by meditation, as positive emotion brought about by meditation helps address various mental problems like social anxiety disorder, post-traumatic stress disorder (PTSD), anxiety, and depression. Overall, it seems to have some impact in all health areas. However, the magnitude of its effect is not known. More diverse and detailed studies should yield more beneficial clinical outcomes.

## Introduction and background

Everything in our daily life starts from one essential thing known as our thoughts. The most important question that needs answering is how much our thoughts impact our everyday life. What changes can we bring about if we can change it? "Mindfulness," also known as meditation and reflection, is the relationship between internal and environmental consciousness [1]. When studied about meditation in the light of neuroscience, our brain indeed has a system through which we can get rid of maladaptive thoughts and restructure our brain [1]. Magnetic resonance imaging (MRI) scans have pointed out that meditation leads to widespread changes in the brain along with the activation of emotional and cognitive centers of the brain [2]. Meditation has shown promising results with age-related brain aging in younger and middle-aged individuals and improved various brain functions, including cognition and other executive functions [3].

It is observed that constant reinforcement of happy thoughts is good for the well-being of both mental and physical health [4]. Moreover, positive reviews positively impact our immune health by reducing interleukin six (IL-6) levels [4]. Such changes at a biological level mean our thoughts have some effect on our physical system. It might be possible that other body systems have a similar impact on our body, and mindfulness can help us yield those hidden gems in our biological system. As seen in the effect on our immune system, our genes are also affected. Effective meditation has had changes in telomerase shortening, which means the aging process can be delayed, as the longevity of our cells tends to increase with appropriate telomerase regulation [5]. The best thing about our brain is that it is a highly complex and neuroplastic structure; however, its scope and diversity are yet to be determined, and how much energy and possibility is there remains a mystery [6]. Is it possible for the way we think to become our reality, and if it is confirmed by memorizing and rehearsing our health, will we be able to change it?

This literature review will shed some light on how mindfulness and our thinking pattern are vital components of our health. With the help of studies, we will decipher if meditation or mindfulness is responsible for significantly changing mental and physical states. This review will also discuss if any changes are occurring at the genetic level or if any changes are observed in our immune system or our inflammatory markers.

## Review

**Figure 1:**
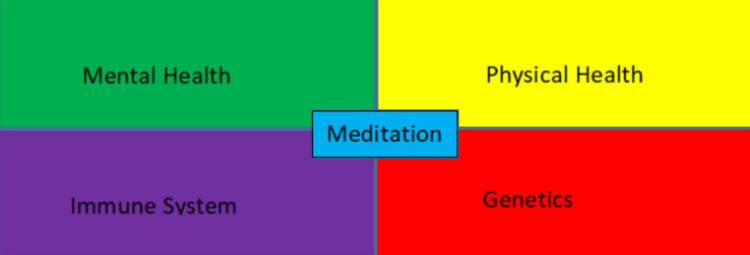
Meditation and its influence on different areas of health The image is the author's creation.

This section will focus in-depth on the various aspects of meditation, and through studies, we will see if there is any proven benefit to the mental and physical health of people who meditate. Furthermore, we will see if there are any fruitful benefits at the genetic or immunological level due to meditation. Figure 1 below shows the areas of health to be discussed that are influenced by meditation.

 

 Meditation and its effect on the immunologic and genetic system

Several studies should be conducted to conclude meditation's impact on our immune health. We looked at a randomized control trial (RCT) done on 1062 participants, and various areas of our immune system were evaluated. It showed that there was a decrease in nuclear factor kappa b (Nf-kb) and C-reactive protein (CRP) levels. An increase in the level of the cluster of differentiation four plus t (CD4+T) cells was seen as an effect of meditation [7]. No effect was there in interleukin one, interleukin six, interleukin eight, interleukin ten, interferon-gamma, influenza, immunoglobulin A (IgA), and immunoglobulin G (IgG), respectively [7]. The study showed some impact on the immune system, but no effect is seen on the interleukins and immunoglobulins. To further assess our immune system, we looked at another RCT. The trial included 26 studies, and different mind-body techniques (MBT) were used, which included yoga, meditation, taichi, and qigong.

In the second study, the results were very similar to those seen in the previous study; no difference was seen in IL-6 levels reduction in the level of Nf-kb. Mixed results were seen when it comes to CRP levels, half of the studies showed no change, whereas half of the studies showed a decreased level of CRP [8]. These mixed results seen in the second study for CRP were probably because of the low power of the study due to a few limitations. For example, the sample size used for the analysis was minimal, the follow-up period was very small, and the inflammatory states of the patients might have contributed to the bias. However, still, the results were close to the previous study. To further strengthen our results, another analysis with 34 studies published in 39 articles with 2219 participants was performed. It included the same mind-body training as seen in the previous survey (meditation, yoga, Tai chi, and qigong). The results are looked at between seven to 16 weeks, and they showed that CRP levels reduced minor non-statistically significant changes seen in IL-6. Antiviral immune responses in natural killer cells, tumor necrosis factor-α, and CD4 count were negligible. However, the immune response to the viral vaccine was observed through mindfulness [9]. As seen in the studies, we can conclude that there is a decrease in inflammatory mediators. No significant change is seen in interleukin. However, the intensity and the clinical impact of mindfulness on our immune system are not known; further studies might help in figuring out its clinical implications. Table 1 below compares the two studies.

**Table 1:** Comparison of immune modulators in both studies IL6, interleukin six; CRP, C-reactive protein; Nf-kb, nuclear factor kappa B; CD4, clusters of differentiation four

Immune modulators	Study 1	Study 2
IL6	No change	No change
CRP	Reduced	Mixed results
Immunoglobulin	No change	Not mentioned
Nf-kb	Reduced	Reduced
Interferon gamma	No change	Not mentioned
CD4 cell count	Increased	Not mentioned

The immune system is not the only system affected by meditation; some studies suggest that meditation affects the length of telomeres [7]. Telomeres are proteins that protect our chromosomes from cell death; increased psycho-social stress increases cell death due to telomere shortening. Still, meditation has been known to increase healthy cell aging due to better regulation of the telomere length [10]. So far, out of 19 studies, two cross-sectional studies showed an increase in telomere length, and nine out of 11 studies showed a telomerase-related increase in telomere length. When the survey of the telomerase-related increase in telomere length is seen for increased duration and intensity, only two out of the nine studies showed a telomerase-related increase in telomere length, and the remaining studies showed no significant change [11].To further verify our conclusion, in another study, 70 trained meditators were selected from meditation training centers from different parts of the island, and only 30 subjects were included in the study after screening; likewise, 30 age- and gender-matched non-meditators were used as the comparison group, and the results showed that the group with meditators showed better quality of life; moreover, the telomerase level was increased in the group of people who meditated compared to non-meditators [11]. These studies show that meditation increases the telomere length and telomerase level, increasing cellular life by preventing telomere shortening. The telomere effect is not the only effect of meditation on our genes, as meditation affects cells in our system at an epigenetic level [11]. When observed, it was seen that meditation reduces methylation of tumor necrosis factor genes related to psycho-social stress in females; moreover, a decrease in methylation of cells involved in immune metabolism was also seen, which would help in enhancing the immune system [11]. The widespread immunological and genetic effects of meditation show how deeply routed the products are, as our immunity and genetics are considered to be a vital part of our overall health, and affecting both systems can be very fruitful in modern medicine.

Meditation and its clinical benefits on physical health

This section will focus on the effects of meditation on different diseases and whether including such practices in your treatment will be of any clinical benefit. One of the widespread multi-factorial diseases is hypertension. Hypertension is usually considered to be caused by poor lifestyle factors and increased sensitivity of the autonomic system, and one of the most important factors is stress. Meditation is a great way to reduce the harmful effects of hypertension [12]. A systematic review and meta-analysis of RCTs were conducted to assess the impact of meditation on systolic and diastolic blood pressure. Six studies were included, which showed a statistically significant effect on diastolic blood pressure reduction compared to the control; on the other hand, the systolic blood pressure results were statistically significant at a very marginal level.

Moreover, those people who were on anti-hypertensive medication showed a better influence on systolic blood pressure with meditation compared to those without a prescription [13]. Another study was done in Korea. This time both hypertensive and diabetic patients were selected as subjects and the effect of education classes (controls) versus brain-education-based meditation (BEM) was studied. In this study, we looked at 48 patients with either diabetes or hypertension. They were divided into two groups, with either BEM or education classes, and the study was conducted for eight weeks in the Ulsan Junggu Public Health Center. Then we looked at levels of glutamic-oxaloacetic transaminase, serum glutamic-pyruvic transaminase, gamma-glutamyl transpeptidase, high-density lipoprotein (HDL), low-density lipoprotein (LDL), expression of inflammatory genes, and reports on physical and mental health were determined. There was a significant decrease in LDL level (13.82; p<0.05) expression of inflammatory genes (0.3-, 0.5-, and 0.2-fold change for Nf-kb 2, v-rel reticuloendotheliosis viral oncogene homolog A (RELA), and interleukin one B (IL1-B), respectively, P<0.05); however, no difference was seen in the remaining biochemical markers including HDL [13]. The results are shown in table 2 below. As discussed, meditation can be a valuable adjunct to other pharmacological interventions, as it has shown some benefits in diabetic and hypertensive patients. Meditation has also shown benefits in diseases like fibromyalgia [14]. The physical effects of meditation should be further studied under different conditions to see the diversity of its impact to see which diseases it affects the most.

**Table 2:** Influence on biochemical markers in diabetic and hypertensive patients due to BEM LDL, low-density lipoprotein; HDL, high-density lipoprotein; Nf-kb, nuclear factor Kappa B; RELA, v-rel avian reticuloendotheliosis viral oncogene homolog A; BEM, brain educated meditation

Biochemichal marker	Effect	Statistical analysis
LDL	Decrease	13.82 P<0.5
HDL	No change	Not mentioned
Nf-kb	Decrease	0.3- P<0.5
RELA	Decrease	0.5- P<0.5
IL1-B	Decrease	0.2 fold P<0.5
Glutamic-oxaloacetic transaminase	No change	Not mentioned
Glutamic-pyruvic transaminase	No change	Not mentioned
Gamma-glutamyl transpeptidase	No change	Not mentioned

Further studies were conducted to check the effect of systolic and diastolic blood pressures on meditation in 2007, which were considered to be a high-quality study in which the sample size was between 37 and 106, as discussed below. The subjects were tested after three months for the meditative intervention; it was seen that there was a 4.3 mm reduction in systolic blood pressure and a 3.11 mm decrease in diastolic blood pressure with a 95% confidence interval (CI) of -6.02 to -0.57 and -4.22 to -3.06, respectively. The result indicated that meditation was four times more effective in reducing blood pressure compared to health education [15]. It has also been seen that meditation has also shown benefits in chronic inflammatory conditions, including asthma and rheumatoid arthritis [16,17]. A systematic literature search was done to see the effect of mindfulness, yoga, and meditation on rheumatoid arthritis, and data from different databases were taken. Out of 1527 studies, only 23 studies were carried. It was interesting to see the effect it has on people who are depressed and the impact on symptoms and disease markers. The results showed that people who meditated had better outcomes, especially those who were depressed. It was also seen that patients felt better subjectively, but there was no difference seen in disease markers like CRP or active disease [17]. The studies discussed so far show that meditation has proven beneficial to some extent to the physical health of the patients.

Meditation and its clinical benefits on mental health

This section will discuss the benefits that can be attained from meditation on our mental health. Meditation is derived from the Buddhist culture, where they believed that "Sukh" or happiness is something that can be achieved by immersing into the nature of reality and by focusing on the present [4]. It has also been shown that meditation increases happiness by explicitly increasing the positive emotional response within the human body [4]. The question remains whether this positive emotional reinforcement affects our mental health clinically. We studied the effect of meditation on mental health. A pilot study was done to check the efficacy of a smartphone-based app to check mood symptoms in cancer patients following meditation. There were several different scales used to assess mood symptoms, and the reading was taken at baseline and an interval of two weeks. There were 35 participants in the study, of which 18 were controls, and 17 were doing meditation. The results revealed a proportional increase in the meditation duration and the improvement of mood symptoms.

Moreover, one of the scales reported improvement in sleep, focus, and mood in the meditation group compared to the intervention group. It showed improved mood symptoms following meditation [18]. The effect of meditation on mental health is not only restricted to minor signs of anxiety but has also been shown to decrease the impact of suicidal thoughts and behavior. A systematic review was done in December 2020 to check whether meditation affects suicidal thoughts and behavior. A total of 14 studies were included from various databases, and all of them showed a reduction in suicidal behavior and ideas, especially those who had major depressive disorder [19].

In today's world, a very under-spoken problem present in our society, which is also considered a global concern nowadays, is "loneliness" [20]. A study was conducted to test whether meditation helps develop social relationship processes. In this study, meditation skills like monitoring of present moment and acceptance might help in improving social relations. The intervention was smartphone-based and included training in monitoring plus approval, monitoring only, and active control in the present moment and acceptance. There were 153 randomly assigned subjects to 14 different smartphone devices, and the intervention was conducted for three days before and after the intervention. Ambulatory measurements of loneliness and daily social contact were obtained. The results showed a 22% percent decrease in loneliness in monitoring plus acceptance compared to monitor only and control training; moreover, it increased two or more interactions each day and one more personal interaction compared to the active-only and monitoring-only groups [20].

As seen by the results, both meditation skills help improve social contact with people and decrease loneliness, which might help improve overall mental health. This study also makes us question if social contact increases with meditation; it might also have a positive effect on problems like social anxiety disorder. It is believed that social anxiety disorder results from negative self-belief, emotional disturbances, and attention biases about yourself [21]. Does meditation affect social anxiety disorder? A study was conducted in which 16 participants were included, and functional MRI scans were obtained while reacting to negative self-belief and also performing a mindful breath-focused emotional regulation. Fourteen participants finished the study, and the results showed that conscious attention led to reduced amygdala activity; decreased anxiety, depression symptoms, and self-esteem; and decreased negative symptoms [21]. It is seen that mental aberrations are something that can be balanced through meditative practices.

**Figure 2:**
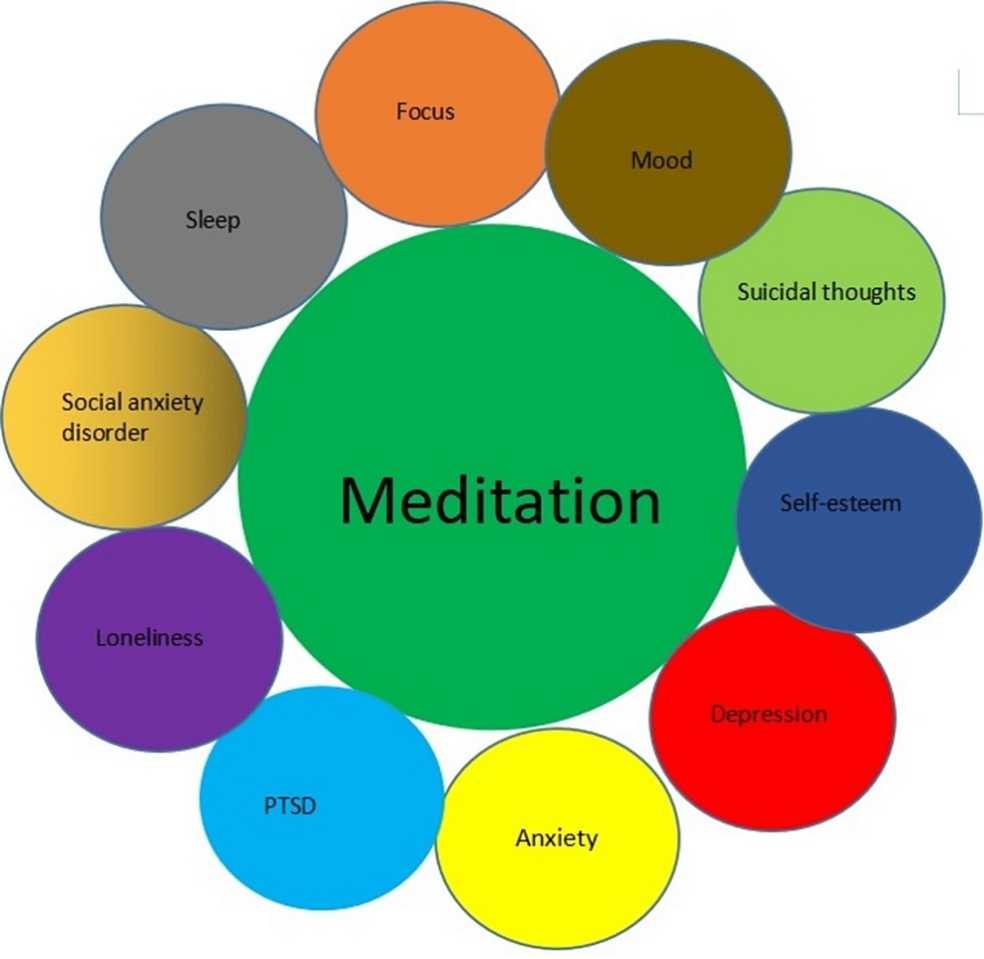
Meditation and its effect on mental health PTSD, post-traumatic stress disorder The image is the author's creation.

Given the above, we can say that meditation has been helpful in various mental disorders; when discussing mental disorders, 7-8% of the people in the united states have post-traumatic stress disorder (PTSD), and they have residual symptoms following the trauma that needs to be treated. It has been seen that meditation and yoga practices have been effective in alleviating those symptoms [22]. If you look at meditation practices on a broader spectrum, it appears as if meditative practices have an overall positive impact on our health. It affects different aspects, including immunology, genetics, and physical and mental health, as discussed. Its effect on the body should be addressed and investigated in greater detail to see if its impact on a disease is good enough to incorporate it as an adjunct to the standard treatment. The problem in such practices could be in their application of how someone meditates and how much effect it has on the disease; moreover, the experiments might also require an increased sample size to come to a suitable conclusion. The illness that can be tested is also limited; on the whole, more studies with more diverse diseases should be done to conclude their efficacy and their certainty for it to be established clinically. Figure 2 below shows the aspects of mental health influenced by meditation.

Limitations

The studies discussed under the topic could have been more precise and accurate if they had filters specific to age, gender, and ethnicity. The studies could have had an increased sample size for increased credibility. The meditators used in the study were not professionals, who could have also been the difference in making the article more precise.

## Conclusions

The health benefits of meditation discussed in this article have positively affected the immune system and genetics. Mindfulness has shown an improved anti-inflammatory response and healthy aging by appropriate telomerase regulation. There are also fruitful benefits seen in physical and mental health, which are positive. Further studies with a larger sample size are needed to determine the magnitude of its effect. As shown by the studies, the diseases in which meditative practices are currently used and are helpful should be continued and recommended. For reflective practice to have clinical benefit, the diversity of the studied diseases should be increased to provide more information about its effect. Moreover, there should be trained professionals who should teach people how to meditate to make its product more pronounced and accurate. More research should be conducted on the topic in the future to have more clinical benefits.
